# Non-Intrusive Load Monitoring Model Based on SimCLR and Visualized Color V-I Trajectories

**DOI:** 10.3390/s26041230

**Published:** 2026-02-13

**Authors:** Tie Chen, Youyuan Fan, Liping Li, Jie Xu, Yifan Xu, Huixia Gan

**Affiliations:** 1 College of Electrical and New Energy, China Three Gorges University, Yichang 443002, China; chent@ctgu.edu.cn (T.C.); 202208580121302@ctgu.edu.cn (Y.F.); 202308580121112@ctgu.edu.cn (L.L.); 202308580121266@ctgu.edu.cn (J.X.); 202308580121270@ctgu.edu.cn (Y.X.); 2 Hubei Provincial Key Laboratory for Operation and Control of Cascaded Hydropower Station, China Three Gorges University, Yichang 443002, China

**Keywords:** non-intrusive load monitoring, contrastive learning, adversarial learning, domain adaptation, cross-domain recognition

## Abstract

**Highlights:**

**What are the main findings?**

- A novel self-supervised framework integrating SimCLR with adversarial domain adaptation effectively aligns cross-domain feature distributions using visualized color V-I trajectories.
- The proposed model achieved an F1-score of 0.9498 with only 10% labeled target data, surpassing the performance of supervised models trained on 30% data.

**What are the implications of the main findings?**

- Integrating adversarial mechanisms into self-supervised learning significantly mitigates domain shift challenges, ensuring robust appliance recognition across diverse household environments.
- The method drastically reduces dependence on manual data annotation, offering a cost-effective and scalable solution for deploying non-intrusive load monitoring systems in smart grids.

**Abstract:**

Current non-intrusive load monitoring (NILM) methods rely on large amounts of labeled historical data and face domain shift issues, which limits the application of deep learning models in practical scenarios. To this end, this paper proposes a SimCLR-ADA-LM framework based on visualized color V-I trajectories. Initially, unlabeled load data from the source domain (PLAID) and target domain (WHITED) are converted into RGB color V-I trajectories and input into the model. The framework enhances intra-class aggregation through contrastive learning and achieves inter-domain feature alignment via adversarial training between the encoder and the domain discriminator to obtain domain-invariant features. Subsequently, the model is fine-tuned using a small amount of labeled data from the target domain to achieve load identification. Ablation and comparative experimental results demonstrate that the proposed model exhibits superior performance advantages over conventional models in cross-domain identification tasks. Furthermore, it maintains significant learning efficiency and recognition robustness even under conditions of limited labeled data.

## 1. Introduction

Under the dual pressure of energy shortages and global warming, energy management, as well as energy conservation and emission reduction, has become a global consensus [1]. As a cost-effective technology for analyzing building energy consumption, non-intrusive load monitoring (NILM) requires only a single sensor deployed at the main service entrance of a building to obtain the operating status and energy consumption information of individual appliances [2,3,4]. While protecting user privacy, NILM enhances the demand-side management of energy suppliers and guides end-users to improve their electricity usage habits, potentially achieving energy savings of approximately 10% [5,6,7].

Although deep learning has significantly improved the recognition accuracy of NILM, its underlying “independent and identically distributed” assumption regarding data limits the cross-domain transferability of these models [8]. In practical applications, substantial differences in appliance models, user behaviors, and environmental noise across different households lead to a severe domain shift between the source and target domains [9,10]. Consequently, addressing the domain shift problem between different users to achieve highly robust cross-domain load recognition has become a critical challenge that the NILM field must overcome to achieve widespread practical application [11].

Scholars have utilized supervised transfer learning and unsupervised domain adaptation to address this issue. Supervised transfer learning fine-tunes classifier parameters using labeled data from the target domain. Reference [12] achieved appliance and cross-domain migration through generalized modeling with shared network weights. Reference [13] designed a lightweight seq2subseq model capable of adaptively obtaining the optimal window, utilizing power data with similar characteristics to train model parameters via transfer learning, thereby enhancing cross-scenario adaptability. Reference [14] transformed 1D power sequences into Gramian Angular Field (GAF) 2D images and employed transfer learning to accelerate the convergence speed of the ResNeXt network in the target domain. However, first, the dependence on labels increases the deployment cost and technical difficulty of load monitoring devices [8,15,16,17], leading to high annotation expenses. Second, supervised learning highly relies on the “independent and identically distributed” assumption. When a model undergoes deep training on the source domain using large amounts of labels, the network parameters tend to capture source-domain-specific data structures. This leads to excessive coupling between the representative features and the source domain distribution, making it difficult for simple model fine-tuning to reverse performance degradation. When facing the same type of appliances with different feature representations, the model is prone to misjudgment due to intra-class feature shift [18,19]. Consequently, large-scale labeled data for new scenarios are still required for retraining, which limits the model’s scalability and engineering feasibility.

Unsupervised domain adaptation (UDA) technologies, which can reduce the dependence on label costs for cross-domain migration, have become a research hotspot in the NILM field. The core of this approach lies in utilizing statistical discrepancy metrics, reconstruction, or adversarial learning mechanisms to achieve the alignment of cross-domain feature distributions without target-domain labels. Reference [20] achieved distribution alignment by introducing statistics such as maximum mean discrepancy (MMD) to directly minimize the inter-domain distribution distance at the feature level. Reference [21] captured shared cross-domain representations by constructing shared encoders and domain-specific decoders using data reconstruction tasks. In contrast, adversarial-based domain adaptation methods leverage a domain discriminator for adversarial training, exhibiting stronger robustness and transfer potential when processing high-dimensional visual data such as digit recognition and object classification [22,23]. Specifically, Reference [24] introduced a Gradient Reversal Layer (GRL) to construct a Domain-Adversarial Neural Network (DANN), validating its effectiveness in cross-domain image recognition tasks such as handwritten digits (MNIST) and street view numbers (SVHN). Although this method achieves the simultaneous optimization of classification and domain alignment via a shared encoder, it tends to weaken feature representational capability, potentially inducing ‘negative transfer’. Reference [25] proposed the Adversarial Discriminative Domain Adaptation (ADDA) strategy, further extending this mechanism to complex general object image transfer. This strategy employs independent encoders for the source and target domains; however, due to the lack of enforced parameter sharing constraints, accurately aligning target domain features with source domain categories remains challenging, leading to ‘semantic drift’ of category positions within the feature space. Fundamentally, these issues stem from a lack of label-guided global alignment, making it impossible to perceive the intra-class feature evolution of loads. In practical scenarios, influenced by user behavior and appliance models, the feature manifestations of the same type of load across different domains often exhibit “intra-class dispersion” (e.g., the current texture of an air conditioner differs drastically under different modes). In the absence of discriminative guidance, blind global alignment not only fails to reduce the feature distance of the same cross-domain load types but also easily leads to feature space confusion due to misjudgment of category affiliation, resulting in recognition errors much higher than those of supervised schemes [11,12,26,27,28].

Self-supervised learning mines the latent structural features of unlabeled data to generate “instance-level pseudo-labels”, which can provide crucial discriminative guidance for adversarial processes that otherwise lack supervision. Self-supervised learning is primarily divided into two categories: generative and contrastive. Generative self-supervised learning focuses on pixel-level local reconstruction, which has limitations in extracting discriminative semantics for load categories [29,30,31]. Contrastive self-supervised methods learn high-order discriminative representations by measuring the similarity between samples in the feature space [32]. For instance, Reference [33] trained Convolutional Neural Networks (CNNs) by solving “jigsaw puzzle” pretext tasks to capture the global topological features of targets, while Reference [34] introduced a MoCo_v2-based time-series self-supervised method specifically for the NILM field, significantly reducing the reliance on labeled data through a momentum contrast mechanism. In contrast, SimCLR, as a representative framework for visual contrastive learning, demonstrates significant potential for cross-modal adaptation. Reference [35] validated its feasibility in handling one-dimensional time-series data. Reference [36] further highlighted that its core advantage lies in data augmentation strategies, such as random cropping and color jittering. These strategies enable the model to discern the ‘spatial structure’ and ‘texture details’ of the data, thereby extracting deep-level information distinct from temporal features to provide a basis for the identification of complex loads. However, such methods are mostly based on the “independent and identically distributed” assumption, ignoring domain shift issues in cross-household scenarios and showing insufficient generalization when processing non-i.i.d. data.

In terms of feature representation, V-I trajectories effectively capture non-linear distortions and phase shifts of loads, exhibiting higher discriminability than traditional one-dimensional power signals [37]. However, normalized V-I trajectories suffer from the loss of numerical information, making it difficult to distinguish devices with similar trajectories but different power magnitudes [38]. Furthermore, traditional binary V-I trajectories lose critical amplitude and temporal information during pixelation, weakening feature separability. To mitigate these limitations and enhance feature resolution, this study employs color encoding to represent numerical values. Based on the electrical physical properties of loads, three strongly complementary physical parameters—reactive power, the ratio of voltage and current rates of change, and the difference between steady-state and transient V-I trajectories—were selected to deeply enhance trajectory shapes. Through feature visualization techniques, these parameters were mapped to the R, G, and B color channels, respectively, to construct color V-I trajectory images. Specifically, reactive power utilizes geometric differences in V-I trajectories (straight lines for resistive loads, ellipses for inductive loads) [39] to distinguish loads with similar shapes but different physical attributes (e.g., fans versus heaters). The ratio of voltage and current rates of change captures non-linear distortions and switching characteristics, differentiating linear from non-linear devices (e.g., laptops, microwaves) via unique texture patterns [40]. The difference between steady-state and transient trajectories encodes device startup transient dynamics [37], distinguishing devices with similar power but distinct startup characteristics. The resulting color trajectory images contain rich spatial texture information, effectively transforming load identification into an image recognition task and thereby improving the recognition rate of non-linear signals [41,42].

In terms of image recognition, early shallow networks such as AlexNet [43] have limited capacity to capture high-order nonlinear features, while traditional deep-stacked architectures like VGG16 [44] suffer from excessive computational overhead due to parameter redundancy. The ResNet series, by introducing residual modules and skip connections, ensures the robust extraction of multi-scale discriminative features while alleviating the gradient vanishing problem in deep networks, allowing for more precise capture of spatial texture details in color V-I trajectory images. Considering that the feature distribution of V-I images is relatively simple, ResNet-18 can avoid the computational waste and overfitting risks associated with deeper architectures such as ResNet-34 or ResNet-50, effectively balancing model performance and inference efficiency. Therefore, this paper adopted ResNet-18 as the encoder to perform feature mining on the fused image data.

Based on the rationales discussed above, this paper integrated the cross-domain alignment capability of domain adaptation technology with the feature discrimination advantages of SimCLR in image classification by embedding a domain discriminator into the SimCLR framework. By utilizing the SimCLR model to mine highly discriminative semantic features and simultaneously leveraging domain adaptation technology to achieve cross-domain distribution alignment, the proposed method enforces the aggregation of features corresponding to the same load type across domains, ultimately enhancing the model’s cross-domain recognition performance.

In summary, this paper designed and implemented a non-intrusive load monitoring model that integrates visualized color V-I trajectories, SimCLR contrastive learning, and ResNet-18. First, unlabeled samples from the source and target domains are converted into visualized color V-I trajectory images for data preprocessing. These V-I trajectory images are then used as model inputs to mine effective representations for load identification through contrastive adversarial self-supervised training. For the downstream load identification features, a classifier is fine-tuned using a very small amount (1–30%) of labeled samples from the target domain to guide the model in completing specific category mapping. Based on the representations learned through self-supervision, the load identification performance of the model was tested to verify its generalizability. Ablation experiments show that the pre-trained encoder achieved an accuracy of 85.3% in its non-fine-tuned state; experimental results further indicate that when using only 10% of the labeled data, the F1-score of the proposed method was significantly superior to that of traditional supervised methods.

The following are the main contributions of this paper:

1. To address the scarcity of labeled data and poor cross-domain performance in NILM, a self-supervised NILM model based on visualized color V-I trajectories was designed. This model integrates the advantages of contrastive learning and adversarial adaptation to mine “domain-invariant and highly discriminative” features in the absence of labels, achieving high-precision cross-domain load recognition under limited label conditions.
2. A deep feature encoder based on residual networks was designed. Through deep feature mining of color V-I trajectory images, the model effectively extracts domain-invariant features of loads, enhancing its transferability across different datasets.
3. A self-supervised framework integrating contrastive learning and adversarial domain adaptation was established. A parameter update mechanism was designed to independently update the domain discriminator parameters using domain discrimination loss, thereby enhancing its domain distinction capability. Furthermore, a joint objective function comprising contrastive loss and adversarial loss was constructed to update the encoder parameters, utilizing a gradient reversal mechanism to eliminate domain-specific biases.

## 2. Methodology

**Figure 1:**
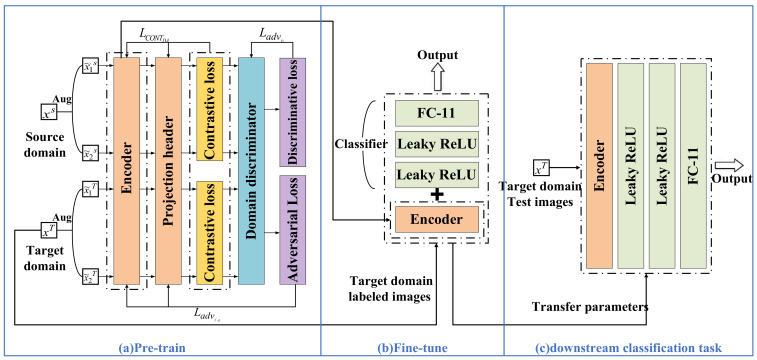
Overall framework diagram.

The contrastive adversarial domain adaptation framework proposed in this paper consists of two stages: pre-training and fine-tuning, as illustrated in Figure 1.

The pre-training stage optimizes the parameters of the ResNet-18 encoder through upstream tasks of contrastive adversarial learning to obtain an encoder with robust representation capabilities. Augmented samples from the source and target domains are mapped to a latent space via the encoder and projection head. Through contrastive loss, the model achieves intra-class feature aggregation and inter-class feature separation, thereby improving the recognition rate of different types of loads. Subsequently, inter-domain differences of the same load types are weakened through a domain discriminator and adversarial learning. The encoder is optimized using adversarial loss to align the distributions of source and target domain data, rendering the source and target domain features indistinguishable. Meanwhile, discriminative loss is utilized to optimize the domain discriminator, enabling it to accurately distinguish between the source and target domains, thereby providing precise discriminative guidance for the encoder’s alignment process.

The downstream task is a classification task aimed at achieving the accurate identification of specific appliances by fine-tuning the pre-trained model. As shown in Figure 1b,c, during the fine-tuning stage, the encoder with deep feature extraction capabilities is retained, and a classifier module, consisting of two Leaky ReLU layers and one fully connected (FC) layer, is connected at the end. Classification and identification of the target loads are achieved through fine-tuning training on the downstream dataset. This section will provide a detailed introduction to each part of the framework.

### 2.1. Upstream Task

The structure of the pre-training framework is shown in Figure 1a, which mainly consists of an encoder, a projection head, and a domain discriminator. First, the encoder codes the augmented data. Subsequently, the projection head maps these encodings into a latent space where contrastive loss is applied. Then, the contrastive loss is calculated to perform feature alignment, and the parameters of the encoder and projection head are updated through backpropagation. Simultaneously, the features are also input into the domain discriminator to calculate adversarial loss and discriminative loss, which are used to update the encoder and the domain discriminator, respectively, to achieve domain alignment. The objective of pre-training is to obtain an encoder capable of outputting high-quality, cross-domain universal features. The implementation of the upstream task mainly includes five parts: color V-I trajectory image data augmentation, pretext task design, encoder construction, domain discriminator construction, and parameter updates.

#### 2.1.1. Data Augmentation

To enhance the model’s representation learning capability, a composite random data augmentation strategy comprising random cropping, color jittering, horizontal flipping, and Gaussian blurring was adopted in this paper. All parameter configurations adhered to the standard settings of SimCLR [45]. The two augmentation processes for the same sample were executed completely independently. The strong randomness of the data augmentation resulted in significant pixel-level discrepancies between the augmented views, ensuring that positive sample pairs retain only device-level semantic features while eliminating pixel-level or positional pseudo-correlations. Furthermore, to prevent the model from acquiring non-semantic discriminative information through sampling distributions, a ‘mixed-domain shuffle sampling’ strategy was employed for training batches. This involves randomly mixing samples from both source and target domains to avoid intra-batch clustering by category or domain.

1. Random Cropping: A region $W_{crop}\times H_{crop}$ is randomly cropped from the original image and resized to a fixed size $W_{fixed}\times H_{fixed}$:

(1) $$I_{crop}=resize\left(crop\left(I,W_{crop},H_{crop}\right),W_{fixed},H_{fixed}\right)$$

2. Color Jittering: Random perturbations of brightness, contrast, saturation, and hue are applied to the $\left(R,G,B\right)$ image pixels with a probability of 0.8:

$I^{\prime }=ColorJitter\left(I;\alpha ,\beta ,\gamma ,\delta \right)$, for example, the brightness transformation is:

(2) $$I_{brightness}\left(x,y\right)=\alpha \cdot I\left(x,y\right)$$

3. Horizontal Flipping: The image is horizontally flipped with a probability of 0.5:

(3) $$I_{flip}\left(x,y\right)=I\left(W-x,y\right)$$

4. Gaussian Blurring: With a probability of 0.5, the image is convolved with Gaussian kernel $G_{\sigma }$, and the blurred image $I_{blur}$ is represented as:

(4) $$I_{blur}\left(x,y\right)=\sum_{u=-k}^{k}\sum_{v=-k}^{k}I\left(x+u,y+v\right)\cdot G_{\sigma }\left(u,v\right)$$

where $G_{\sigma }\left(u,v\right)=\frac{1}{2\pi \sigma^{2}}\exp\left(-\frac{u^{2}+v^{2}}{2\sigma^{2}}\right)$.

#### 2.1.2. Pretext Task Design

Pretext tasks are important strategies for learning data representations using pseudo-labels. In this paper, two pretext tasks—contrastive learning and adversarial training—were designed to drive model training by constructing instance-level and domain-level pseudo-labels, respectively.

The contrastive learning pretext task utilizes “instance labels” between augmented views of samples to construct positive and negative sample pairs, continuously reducing the distance between similar samples in the feature space and automatically generating pseudo-labels based on attributes found in the data [45]. For an input batch containing N images, two random data augmentations are performed on each sample, generating a total of 2N augmented views. Within this batch, two views derived from the same original image constitute a positive pair, while the remaining $2\left(N-1\right)$ views are defined as negative samples. The construction of positive and negative samples is based solely on device category attributes, independent of the source household, thereby avoiding interference from household distribution clues. In the encoding stage, all 2N views are input into a shared-parameter feature encoder $f\left(\cdot \right)$ to obtain high-dimensional feature vectors h, which are then mapped to a latent space z through a projection head $g\left(\cdot \right)$ composed of a two-layer multi-layer perceptron (MLP). This mapping process is defined as:

(5) $$z=g\left(h\right)=W^{\left(2\right)}\sigma \left(W^{\left(1\right)}h\right)$$

In the formula, σ is the ReLU activation function, while $W^{\left(1\right)}$ and $W^{\left(2\right)}$ are the weight matrices of the first and second layers of the projection head, respectively.

The adversarial training pretext task assigns label d=0 to source domain samples and label d=1 to target domain samples. Using the same feature vector h output by the encoder as input, a Gradient Reversal Layer (GRL) is implemented between the encoder and the domain discriminator. During the forward propagation process, feature h is input into the discriminator through the GRL via identity mapping, and the discriminator outputs the predicted probability of the sample belonging to a specific domain. During the backpropagation process, the GRL multiplies the gradient from the discriminator by a negative constant −λ before passing it back to the encoder.

#### 2.1.3. Encoder

**Figure 2:**
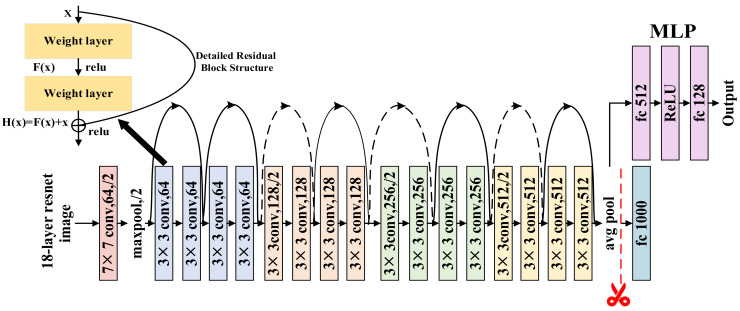
Structure diagram of the feature representation network.

The feature representation network consists of two parts: the encoder and the projection head, the overall structure of which is shown in Figure 2. The encoder is responsible for extracting high-order features from the input color V-I trajectory images, while the projection head maps the feature vectors into the contrastive loss space. The specific design is as follows:

1. Encoder Structure Design

This paper adopted ResNet-18 as the basic encoder architecture. This architecture includes an initial convolutional layer, a max-pooling layer, and four stages of residual mapping groups. For the input color V-I trajectory images, the encoder achieves hierarchical feature extraction through multi-layer convolutions [34]: low-level convolutional kernels primarily capture the edges and fine texture information of the image, while mid-to-high-level convolutions further identify high-order structural patterns such as color distribution and shape contours. The basic residual learning units effectively preserve the original feature information through identity mapping without introducing additional parameters or computational complexity.

2. Mathematical Model of Residual Learning

ResNet [46,47] mitigates the problems of gradient vanishing and performance degradation in deep networks by introducing a skip connection mechanism. The mathematical model of its basic residual unit is defined as:

(6) $$y=F\left(x,\left\{W_{i}\right\}\right)+x$$

In the formula, x and y represent the input and output vectors of the residual unit, respectively; function $F\left(x,\left\{W_{i}\right\}\right)$ denotes the residual mapping that the network needs to learn. When the dimensions of the input and output are inconsistent, dimension matching is performed through linear projection $W_{s}$, and the formula evolves into:

(7) $$y=F\left(x,\left\{W_{i}\right\}\right)+W_{s}x$$

This mechanism ensures that the model maintains training stability while increasing depth, allowing for the more precise extraction of multi-scale discriminative features from the color V-I trajectories.

3. Projection Head Construction

In the feature decoding stage, the projection head adopts a Multi-Layer Perceptron (MLP) structure, consisting of fully connected layers and ReLU activation layers. This module enhances the separability of features in the contrastive learning space through nonlinear mapping while simultaneously mapping feature vectors to a latent space suitable for metric learning, thereby improving the model’s ability to generate discriminative representations for load categories [45].

#### 2.1.4. Domain Discriminator

**Figure 3:**
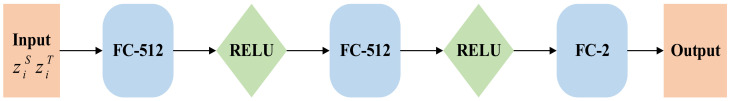
Structure diagram of the domain discriminator.

The domain discriminator is a three-stage lightweight network constructed with fully connected layers and ReLU activation functions, the structure of which is shown in Figure 3. Its modules include the feature projection module: it receives features $z_{i}^{S}$ and $z_{i}^{T}$ from the projection head, maps them into a unified latent space via a fully connected layer, and implements nonlinear transformation through ReLU activation; the deep discrimination module utilizes a fully connected layer (512→512) and ReLU to extract high-order domain-discriminative features, where single-layer nonlinear processing preserves domain difference information while avoiding overfitting; the binary decision module is the final fully connected layer that compresses the 512-dimensional features into a 2-dimensional output, generating discrimination scores for the source/target domains.

An adversarial game based on the binary cross-entropy loss is constructed between the encoder G and the domain discriminator D. The model assigns domain labels to load samples to represent the data source. The domain discriminator D receives the feature vectors extracted by the encoder G and outputs a probability prediction of whether the sample belongs to the target domain. For the domain discriminator, the objective is to minimize discrimination error to accurately distinguish between the source and target domains; meanwhile, the objective of the encoder is to “deceive” the discriminator by generating ambiguous features. Thus, the two constitute a minimax game objective, and the objective function is:

(8) $$\underset{G}{\min}\;\underset{D}{\max}L_{adv}(G,D)=E_{x^{S}}\left[\log D\left(G(x^{s})\right)\right]+E_{x^{t}}\left[\log\left(1-D\left(G(x^{t})\right)\right)\right]$$

The introduced Gradient Reversal Layer (GRL) automatically flips the sign of the gradient from the discriminator loss, causing the encoder to continuously eliminate domain-specific biases during the training process, thereby forming a game. When the discriminator’s predicted probability of the sample source approaches 0.5 (meaning that it is completely unable to distinguish which domain the features come from), the game reaches a dynamic equilibrium. The mathematical expression is:

(9) $$\left\{\begin{array}{l} R_{\lambda }\left(x\right)=x\\ \frac{dR_{\lambda }}{dx}=-\lambda I \end{array}\right.$$

In the formula, x is the feature vector input to the layer; $R_{\lambda }\left(x\right)$ is the identity mapping output during forward propagation; λ is the gradient reversal coefficient; Ι is the identity matrix.

#### 2.1.5. Parameter Update

**Figure 4:**
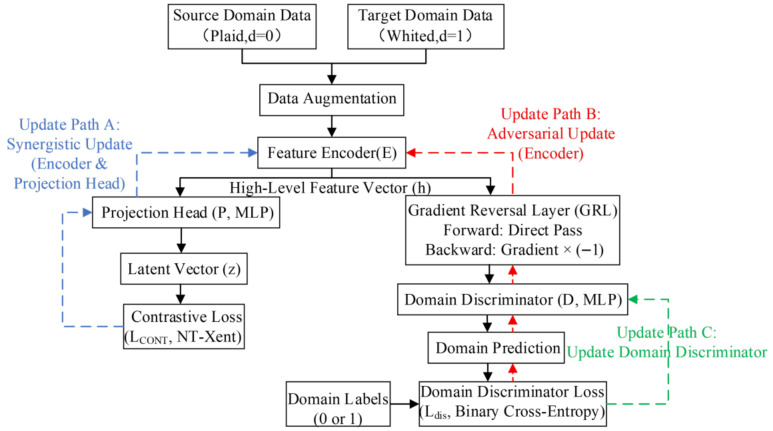
Parameter update flowchart.

Optimization of the model parameters is a process of multi-task collaborative training. As shown in Figure 4, in a single training iteration, after the data flow through the network to calculate the loss, the parameters of the encoder, projection head, and domain discriminator are collaboratively updated through three different gradient backpropagation paths (Path A, B, and C).

1. Parameter Update of the Encoder (Joint Optimization)

The encoder E is the core of the entire architecture. Its parameter updates are jointly driven by the contrastive learning loss and the adversarial training loss, aiming to extract universal features that are both highly discriminative and domain-invariant.

First, in update Path A, the model utilizes a contrastive loss function to guide the encoder in learning discriminative representations. In a batch containing N samples, 2N views are generated through data augmentation. For a pair of positive samples $\left(i,j\right)$, their feature vectors are mapped to $z_{i}$ and $z_{j}$ through the projection head, and the Normalized Temperature-scaled Cross Entropy Loss (NT-Xent) [1] is employed for calculation. This aims to maximize the cosine similarity of positive sample pairs while minimizing their similarity with the other $2\left(N-1\right)$ negative samples in the batch. The loss for a single positive sample pair $\left(i,j\right)$ is defined as follows:

(10) $$l\left(i,j\right)=-\log\frac{\exp\left(sim(z_{i},z_{j})/\tau \right)}{\sum_{k=1}^{2N}1_{\left[k\neq i\right]}\exp\left(sim(z_{i},z_{k})/\tau \right)}$$

In the formula, $s_{i,j}=\frac{z_{i}^{T}z_{j}}{\left\|z_{i}\right\|\left\|z_{j}\right\|}$ represents the cosine similarity, τ is the temperature parameter, and $1_{\left[k\neq i\right]}\in \left\{0,1\right\}$ is an indicator function that takes the value of 1 when k≠i and 0 otherwise. According to Formula (10), the total loss function for all positive sample pairs in the minibatch is calculated as:

(11) $$L=\frac{1}{2N}\sum_{k=1}^{N}\left[l\left(2k-1,2k\right)+l\left(2k,2k-1\right)\right]$$

Finally, the cumulative NT-Xent for each domain is calculated using the following formula:

(12) $$L_{CONT}=L_{CONT_{S}}+L_{CONT_{T}}$$

where $L_{CONT_{S}}$ and $L_{CONT_{T}}$ are the NT-Xent loss values calculated from the source domain images and target domain images, respectively. By minimizing the total loss $L_{CONT}$ of all positive sample pairs in the batch, gradients backpropagate along Path A to collaboratively update the parameters of encoder E and projection head P. This guides the encoder to extract high-order discriminative representations conducive to load type identification through contrastive learning.

Second, in update Path B, the model further optimizes the encoder using reversed gradients generated from the domain discrimination loss. During this process, the encoder’s goal is to generate features capable of “deceiving” the discriminator—that is, maximizing the classification error of the domain discriminator. This is mathematically equivalent to minimizing the negative domain discrimination loss. The gradient originates from the domain discrimination loss $L_{dis}$, as it passes through the Gradient Reversal Layer (GRL) during backpropagation, its sign is automatically flipped (multiplied by a negative constant −λ). This reversed signal is transmitted along Path B to the encoder, forcing the encoder parameters E to update in a direction that blurs the distributions of the source and target domains. The domain discriminator parameters remain fixed while the encoder is being updated.

In summary, the final update objective of the encoder is to minimize the following total objective function:

(13) $$L_{Encoder}=L_{CONT}+\lambda L_{adv}$$

In the formula, $L_{adv}$ is the adversarial loss term (i.e., the reversed discrimination loss), and λ is the hyperparameter used to balance the weights of the two tasks.

2. Parameter Update of the Domain Discriminator (Independent Optimization)

The parameter update of the domain discriminator relies solely on Path C. This module receives the feature h output by the encoder, treats it as input x, and performs binary classification training combined with the ground-truth domain label d. Its loss function is defined using the standard binary cross-entropy:

(14) $$L_{dis}=-{Ε}_{\left(x,d\right)}\left[d\log D\left(x\right)+\left(1-d\right)\log\left(1-D\left(x\right)\right)\right]$$

In this stage, the gradient along Path C is used solely to update the parameters of the domain discriminator, while the encoder parameters remain fixed. The optimization objective of the discriminator is to minimize $L_{dis}$, thereby improving its accuracy in distinguishing the source of the features (source domain or target domain).

Through the aforementioned mechanism, the encoder and the domain discriminator are updated alternately during the training process, forming a dynamic game between “the encoder attempting to merge domain differences” and “the discriminator attempting to identify domain differences”. Ultimately, when the model converges, it is able to learn high-quality feature representations that contain both load semantic information and eliminate domain bias.

### 2.2. Downstream Tasks

After the pre-training is completed, the encoder is migrated to the fine-tuning model, as shown in Figure 1b. A new fully connected layer is added after the encoder as a classifier. For the downstream load classification task, a small portion of labeled data from the target domain training set is used to fine-tune both the encoder and the classifier.

The SGD optimizer is used to optimize the parameters of the classifier, with the initial learning rate set to 30. A Multi-Step Learning Rate decay (MultiStepLR) is adopted to dynamically update the learning rate, decaying it to one-tenth of its previous value at each update. This enables the network to converge rapidly while better reaching the optimal solution. After fine-tuning is completed, the parameters of the entire fine-tuned model are saved to implement the downstream load multi-objective classification task, as shown in Figure 1c.

## 3. Evaluation Metrics

A confusion matrix [40,48] was adopted to visualize the identification results. The evaluation metrics in this paper include Accuracy (Acc), Precision (P), Recall (R), and the F1-score. The calculation formulas for each metric are as follows:

Accuracy: The proportion of the number of correct identifications to the total number of identifications.

(15) $$A=\frac{TP+TN}{TP+TN+FP+FN}$$

Precision: The proportion of the number of times a device is correctly identified to the total number of times it is identified as that device.

(16) $$P=\frac{TP}{TP+FP}$$

Recall: The proportion of the number of times a device is correctly identified to the total number of its actual occurrences.

(17) $$R=\frac{TP}{TP+FN}$$

F1-score: The harmonic mean of precision and recall.

(18) $$F1=\frac{2\times P\times R}{P+R}$$

## 4. Case Study

All case studies were conducted on the Windows 11 operating system using PyTorch 2.0.1 and CUDA 12.3. The computing platform configuration consisted of an Intel Core i5-13400 CPU and an NVIDIA GeForce RTX 4090 GPU. To eliminate the influence of random initialization on the experimental results and ensure statistical reliability, all experiments in this paper were independently repeated five times with fixed random seeds, and the average value of the five runs was adopted.

### 4.1. Datasets

This paper selected two publicly available high-resolution load datasets, PLAID [49] and WHITED [50], to validate the proposed method. The PLAID dataset was collected from 55 households in the United States and contains a total of 1074 samples covering 11 categories of appliances, with a sampling rate of 30 kHz for voltage and current data. The WHITED dataset covers 54 categories of appliances across multiple countries, totaling 1259 samples with a sampling rate of 44.1 kHz, offering higher device diversity and cross-regional representativeness.

Considering that cross-domain transfer learning necessitates a consistent label space between the source and target domains, 11 device categories shared with the PLAID dataset were selected from the 54 device types available in the WHITED dataset. (Air Conditioner (AC), Compact Fluorescent Lamp (CFL), Fan (fan), Refrigerator (fridge), Hairdryer (hairdryer), Heater (heater), Incandescent Light Bulb (ILB), Laptop (laptop), Microwave (microwave), Vacuum Cleaner (vacuum), and Washing Machine (washing)). These categories served as common labels for both the source and target domains and were utilized to perform fine-tuning with a limited amount of labeled data in the target domain, thereby accomplishing the cross-domain load identification task.

### 4.2. Data Preprocessing

The methods described in [51,52,53] were adopted to construct V-I trajectories. Subsequently, three physical parameters—reactive power, the ratio of voltage and current rates of change, and the difference between steady-state and transient trajectories—were selected and mapped to the RGB color channels, respectively, to construct color V-I trajectory images. During the preprocessing phase, irrelevant metadata such as household identifiers and collection timestamps are eliminated. Only core features directly related to the physical characteristics of the devices (e.g., voltage, current, and reactive power) are retained, thereby eliminating explicit household-level distribution clues at the source. Simultaneously, the distinct sampling rates of the two datasets (30 kHz and 44.1 kHz) are standardized to a unified level to prevent the introduction of implicit household-related correlations caused by discrepancies in data acquisition conditions.

(1) R-channel (Reactive Power Feature): Reactive power Q is mapped to the red channel, where variations are directly reflected in the geometric shape of the V-I trajectory. The calculation formula is given by:

(19) $$R\left(i_{g},v_{g}\right)=\frac{Q}{S}=\frac{\frac{1}{M}\sum_{m=1}^{M}v_{m}i_{m}}{V_{rms}I_{rms}}$$

In the formula, $R\left(i_{g},v_{g}\right)$ represents the R value in the i_g_-th row and v_g_-th column of the red matrix; S represents the apparent power; $I_{rms}$ represents the RMS value of the current; g = 1, 2, ⋯, n.

(2) G-channel (Non-linear Harmonic Feature): The ratio of voltage to the rate of change of active current is mapped to the green channel to capture the non-linear distortion and switching characteristics of the load. The calculation formula is given by:

(20) $$G_{g}=\arctan\left(\frac{i_{g+1}-i_{g}}{i_{\max}},\frac{v_{g+1}-v_{g}}{v_{\max}}\right)$$

In the formula, $i_{\max}={\left|i\left(t\right)\right|}_{\max}$ and $v_{\max}={\left|v\left(t\right)\right|}_{\max}$ are the maximum values of the absolute current and the absolute voltage within a stable period, respectively.

(3) B-channel (Transient Impulse Feature): The difference between the non-steady-state and steady-state V-I trajectories is mapped to the blue channel to encode the transient dynamic information at the moment of device startup. The calculation formula is given by:

(21) $$B=\frac{1}{Z}\sum_{z=1}^{Z}W_{z}$$

**Figure 5:**
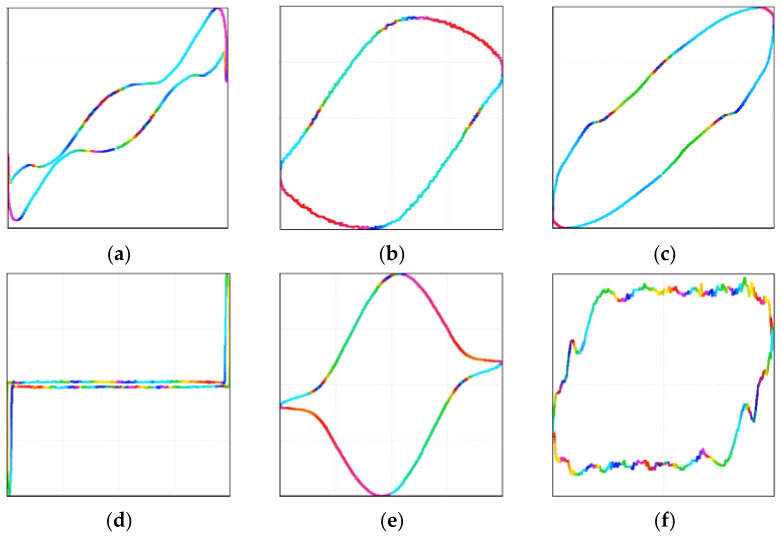
Color V-I trajectory images of different electrical loads: (**a**) AC, (**b**) fan, (**c**) fridge, (**d**) laptop, (**e**) microwave, and (**f**) washing machine.

In the formula, Z represents the number of cycles of the load during the non-stable operation stage; $W_{z}$ represents the value of the corresponding pixel point in the V-I trajectory characteristics of the z-th non-stable stage. Taking some devices in the WHITED dataset as examples, the formed color V-I trajectories are shown in Figure 5:

### 4.3. Case Study 1

**Figure 6:**
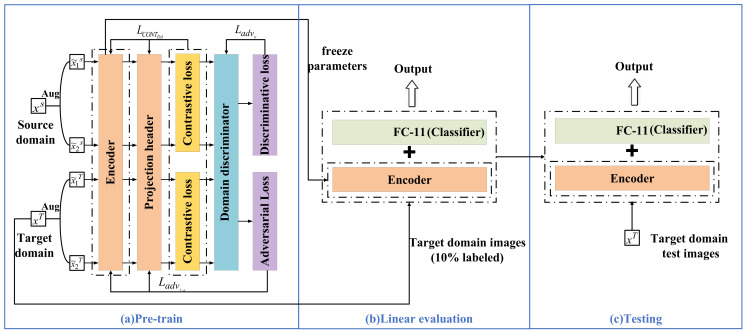
Schematic diagram of the linear evaluation framework.

Case Study 1 follows the linear evaluation protocol to conduct ablation experiments. As shown in Figure 6, the pre-training projection head is removed and the encoder parameters are fully frozen. A single fully connected (FC) linear layer is added at the end as a classifier, and this linear layer is fine-tuned independently under a fixed labeling ratio. This section sets up the following three models:

(1) SimCLR-S: Self-supervised contrastive pre-training is performed only on the source domain. This model serves as the base variant to verify the basic effectiveness of the contrastive learning mechanism in extracting deep load features.
(2) SimCLR-M: Contrastive learning is performed on mixed unlabeled data from the source and target domains without introducing adversarial training. By comparing with SimCLR-S, this model verifies the actual impact of domain adaptation on improving the model’s generalization ability.
(3) Proposed: Adversarial training is integrated into mixed-domain contrastive learning by adding a domain discriminator. Through comparison with SimCLR-M, this model verifies the key role of the adversarial game in aligning cross-domain distributions and extracting domain-invariant features.

Case Study 1 uniformly sets the total number of pre-training samples to 22,000 trajectory images to ensure a fair comparison. Among them, models with domain adaptation (SimCLR-M and the Proposed method) sampled 11,000 unlabeled images from the source and target domains respectively, while the non-domain adaptation model (SimCLR-S) sampled all images from the source domain. During the linear evaluation stage, 10% of the labeled samples from the target domain were randomly selected to train the classification layer; the test set consisted of 300 images per device in the target domain that have never participated in the training process (ensuring consistency between test and validation data). By calculating the classification accuracy on the test set, the feature extraction performance and generalization ability of each ablation model on unknown samples were quantitatively evaluated. The data usage and network structure of each model are shown in Table 1, and the training parameter configurations are shown in Table 2:

Table 3 records in detail the recognition accuracy of different models on the target domain, quantitatively validating the necessity of each core component. First, the SimCLR-S model, which adopts only self-supervised contrastive learning, achieved an accuracy of 68.8%, proving that this module is significantly effective in extracting high-order nonlinear features of loads and establishing a solid representation foundation for subsequent tasks. On the basis of SimCLR-S, the SimCLR-M model, which introduces unlabeled data from the target domain, further improved the accuracy by 6.5% to reach 75.3%; this validates that utilizing target domain samples for pre-training enhances the model’s ability to map the feature distribution of the target domain, thereby achieving preliminary convergence of inter-domain differences and effectively mitigating recognition interference caused by domain shift. Furthermore, the proposed method in this paper achieved a performance leap of 10.0% compared to SimCLR-M (reaching 85.3%) by introducing an adversarial mechanism, directly confirming the critical role of adversarial domain adaptation in aligning cross-domain distributions and extracting universal representations. The experimental results show that the proposed method achieved the highest accuracy across all appliances, with particularly significant improvements in categories with complex nonlinear features such as Fridge, Heater, and Vacuum, systematically validating the superiority of the synergy between contrastive learning and adversarial mechanisms. Furthermore, the results effectively rule out the ‘gradient conflict’ hypothesis often associated with multi-task learning. Specifically, if the optimization objectives of the contrastive loss and the adversarial loss are in conflict, the incorporation of adversarial domain adaptation would typically lead to model oscillation or performance degradation. In contrast, the significant performance gains observed in this study demonstrate that the interaction between the dual loss functions is constructive and synergistic.

**Figure 7:**
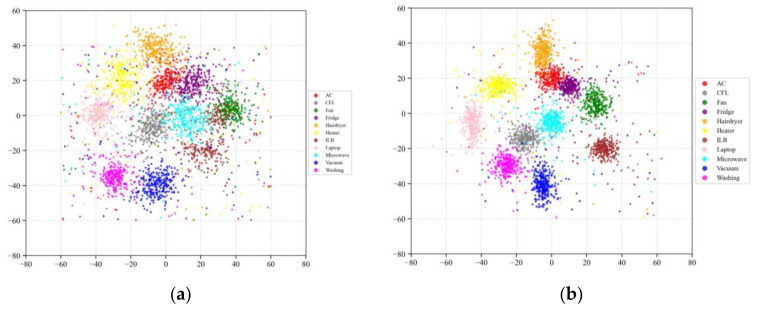
t-SNE visualization: (**a**) SimCLR-M, and (**b**) proposed.

Using SimCLR-M and the proposed method as representative examples, the t-SNE [54] visualizations of feature distributions on the target domain test data are presented in Figure 7. Although SimCLR-M enhanced feature discriminability through contrastive learning and adversarial domain adaptation, certain degrees of class confusion persisted. In contrast, the feature distributions extracted by the proposed method exhibited greater compactness and more distinct inter-class boundaries. This observation corroborates the quantitative improvements presented in Table 3, visually substantiating the model’s enhanced capability in feature discrimination.

### 4.4. Case Study 2

Case Study 2 aims to evaluate the recognition performance of the proposed method on the WHITED dataset under low labeling ratios of 1%, 10%, 20%, and 30% in the target domain. By conducting a horizontal comparison with the following four classic supervised learning models, this study verified the performance advantages of the proposed scheme in label-scarce scenarios:

(1) AlexNet: Serves as a representative of shallow convolutional architectures to provide a baseline performance benchmark.
(2) VGG16: Adopts a classic deep stacking structure to verify recognition stability under conventional deep feature extraction.
(3) resnet-18: Ensures robust extraction of multi-scale discriminative features through residual modules and skip connections, serving as an important benchmark for measuring residual network performance.
(4) SE-ResNet: Incorporates a channel attention mechanism into the residual structure, representing a current convolutional model scheme with advanced perception capabilities.

The Adam optimizer is employed for training, and the parameters for pre-training and fine-tuning, along with the data usage details, are listed in Table 4 and Table 5, respectively. To mitigate the issue of class imbalance among device categories, a stratified random sampling strategy was adopted. Specifically, 2000 unlabeled samples were selected from each device category across both datasets for self-supervised contrastive adversarial pre-training. Furthermore, for the WHITED dataset, 500 samples per category were allocated as the validation set, while 1000 samples served as the test set. To further evaluate model performance under few-shot annotation conditions, subsets corresponding to 1%, 10%, 20%, and 30% of the data were randomly sampled from the pre-training set of each device category in the WHITED dataset for fine-tuning.

The F1-score effectively measures the recognition performance of the models, and the F1-scores for each model under different labeling ratios are shown in Table 6. Under the extremely low labeling ratio of 1%, the supervised models—AlexNet, VGG16, ResNet-18, and SE-ResNet—all yielded F1-scores below 0.34, as they struggle to extract generalized features from limited samples. In contrast, the proposed method achieved a score as high as 0.8449 by leveraging the prior information captured during pre-training, representing a significant lead. When the labeling ratio increased to 10%, the F1-score of the proposed method leapt to 0.9498, an accuracy level that already surpassed the recognition performance of traditional supervised models at a 30% labeling ratio. This indicates that the proposed scheme achieves more precise load recognition with lower data dependency, meeting the requirements for recognition accuracy in practical engineering. In tests across different labeling scales, the proposed method consistently demonstrated a significant performance advantage, leading SE-ResNet by 27.9%, 9.49%, and 6.6%, respectively. These results fully validate the superior stability and generalization robustness of the contrastive adversarial strategy in label-scarce cross-domain recognition tasks.

**Figure 8:**
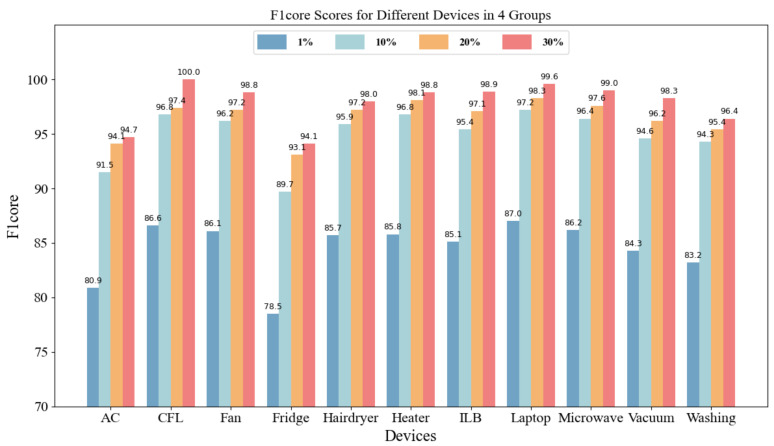
F1-scores of the proposed method for each appliance under different labeling ratios.

Figure 8 illustrates the F1-scores of the proposed method for various types of appliances under different labeling ratios. The results indicate that the proposed method extracts high-quality features even under low-labeling conditions, and its performance improves steadily as the amount of labeled data increases, validating its effectiveness and robustness in appliance recognition tasks. Even at a mere 1% labeling ratio, the F1-scores for all appliances were no lower than 78.5%, with CFL, Fan, Laptop, and Microwave exceeding 86%, demonstrating strong recognition capabilities. As the labeling ratio rose to 10%, all appliances except for the Fridge surpassed 90%. At a 30% labeling ratio, the F1-scores for all appliances exceeded 94%, with CFL reaching 100%.

**Figure 9:**
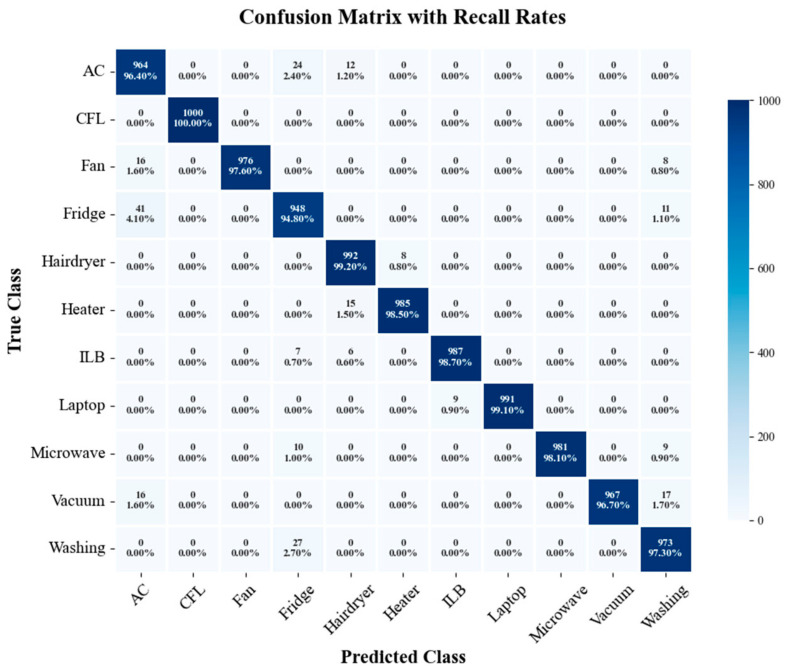
Confusion matrix of the proposed method at a 30% labeling ratio.

**Figure 10:**
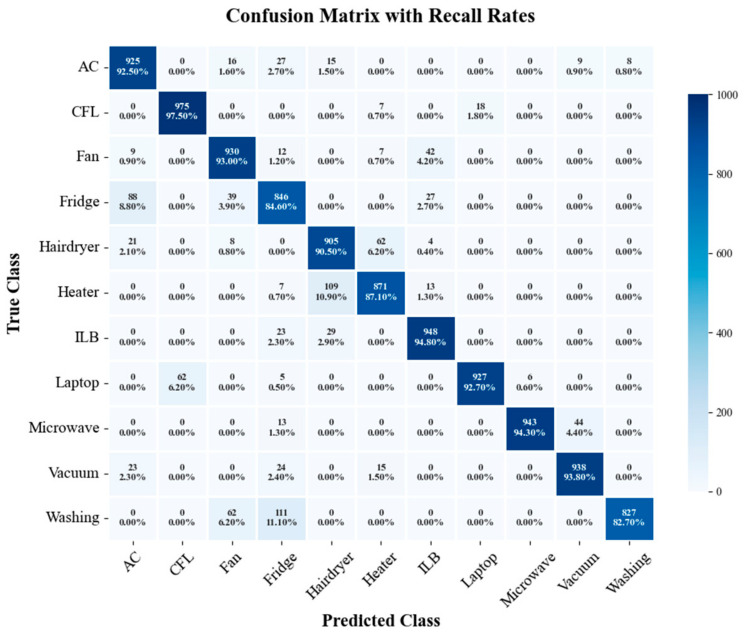
Confusion matrix of the SE-ResNet method at a 30% labeling ratio.

To further evaluate the model performance in appliance recognition tasks, Figure 9 and Figure 10 present the confusion matrix heatmaps of the proposed method and SE-ResNet trained with 30% labeled data, respectively. The numerical data in the figures represent the count of misclassified appliances, while the percentages denote the recall rates. Overall, the proposed method extracts appliance features more effectively during the pre-training phase, with recall rates for most appliances exceeding 97%. In contrast, SE-ResNet exhibited significant misclassifications in categories such as Fridge and Heater, which were easily confused with Washing Machine and Hairdryer. Under the same training conditions, the recognition results of the proposed method were more concentrated with less inter-class confusion, demonstrating stronger feature discrimination and robustness. This further validates the effectiveness of the contrastive learning and adversarial domain adaptation strategy in cross-domain tasks. Furthermore, Table 7 and Table 8 list four evaluation metrics derived from the confusion matrices for both methods at the 30% labeling ratio. The results show that the proposed method outperformed SE-ResNet in terms of accuracy, precision, recall, and F1-score, reflecting a superior overall performance.

**Figure 11:**
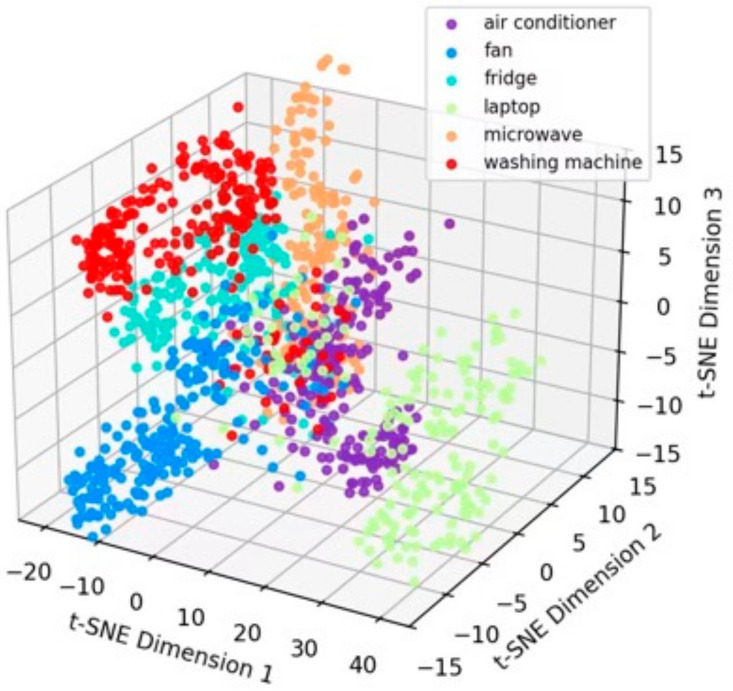
t-SNE visualization of the raw input data.

**Figure 12:**
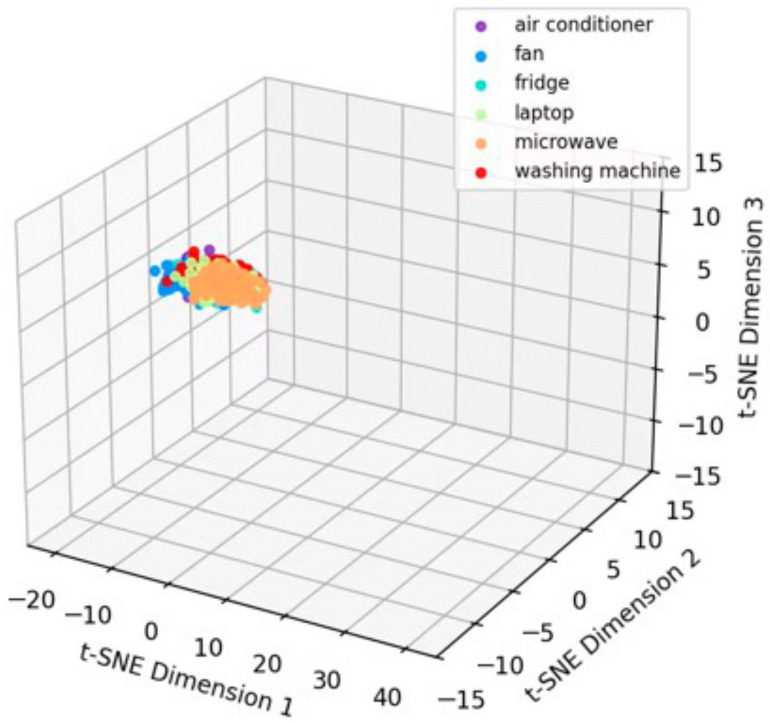
t-SNE visualization of the feature representations extracted by the proposed method.

Taking six devices—Air Conditioner, Fan, Refrigerator, Laptop, Microwave, and Washing Machine—as examples, Figure 11 and Figure 12 utilize the t-distributed stochastic neighbor embedding (t-SNE) algorithm to map the raw input data and the output features of the proposed method, respectively, into a three-dimensional space for visualization. As observed from the figures, the contrastive learning and adversarial domain adaptation mechanisms acquire highly discriminative feature representations leveraging solely unlabeled data, resulting in the distinct clustering of features corresponding to different devices within the three-dimensional space. This substantiates the effective utilization of massive historical unlabeled data by the upstream tasks in the proposed method.

**Figure 13:**
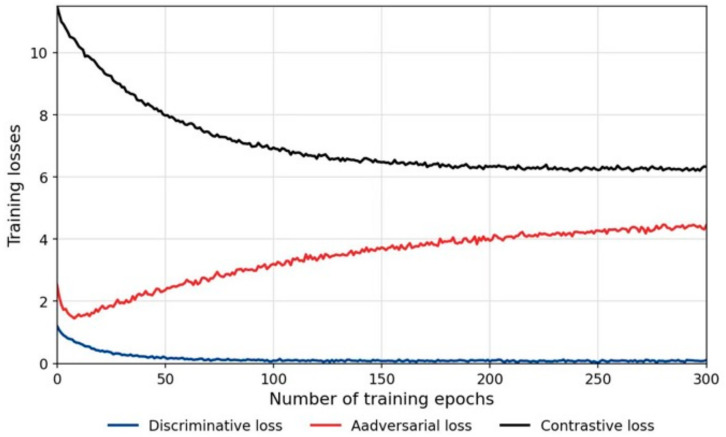
Learning curves of the proposed method on the training data.

Figure 13 depicts the learning curves during the training process, tracking the trajectories of the adversarial loss, domain discriminator loss, and contrastive loss. The results indicate that as the number of iterations increases, all three loss components tend to stabilize, exhibiting no oscillation or divergence attributable to gradient conflicts. This confirms the stability of the training process and demonstrates that the objectives of contrastive learning and adversarial domain adaptation are mutually compatible, achieving constructive synergistic optimization.

Table 9 presents the statistical results of F1-scores obtained from five independent repeated experiments. The data indicate that the sample standard deviations across all labeled data proportions were consistently within 0.2%. This minimal fluctuation reflects the model’s robust stability, demonstrating its ability to effectively mitigate the uncertainty introduced by random initialization and achieve stable convergence.

To comprehensively evaluate the computational cost and potential for engineering application, the model parameters, training duration, and inference latency were tested on an NVIDIA GeForce RTX 4090 GPU, with the statistical results listed in Table 10. As indicated in the table, due to the incorporation of dual-view data augmentation for contrastive learning and the adversarial training mechanism, the offline pre-training time of the proposed method was 4.1 h, which was higher than that of the traditional supervised learning model, ResNet-18. However, in practical applications of non-intrusive load monitoring (NILM), online inference efficiency is the critical factor determining the feasibility of deployment on edge devices. Since the projection head and domain discriminator used for auxiliary training are removed during the inference phase of the downstream classification task, the actual inference latency of the proposed method was only 4.5 ms per sample, comparable to the lightweight ResNet-18 (4.1 ms). This demonstrates that while the proposed method incurs higher offline training costs, it does not compromise real-time online identification capabilities, thus establishing a foundation for future practical engineering deployment.

### 4.5. Parameter Sensitivity Analysis

To validate the robustness of the model to key hyperparameters, a parameter sensitivity analysis was conducted on the WHITED target domain. The experiments specifically analyzed the impact of the contrastive learning temperature coefficient, the gradient reversal coefficient in adversarial training, and the batch size on the F1-score.

The temperature coefficient τ regulates the focus of the contrastive loss on hard negative samples, while λ controls the weight of domain adaptation within the total loss function. As presented in Table 11, the model was evaluated with τ set to 0.05, 0.1, 0.5, and 1.0, respectively. Optimal performance was achieved at τ=0.1 (F1 = 0.9498); furthermore, the F1-scores consistently remained above 0.91 within the range of 0.05 to 0.5, exhibiting significant stability. Similarly, as λ varied from 0.1 to 1.0, the fluctuation in model accuracy was negligible. This demonstrates that the proposed synergistic contrastive-adversarial mechanism effectively reduces the model’s sensitivity to individual hyperparameters.

To balance the dependency of contrastive learning on the number of negative samples with GPU memory constraints, performance was compared across batch sizes of 64, 128, and 256. Experimental results indicate that the model achieved the fastest convergence and optimal performance with a batch size of 128. Although increasing the batch size to 256 provided a larger pool of negative samples, the marginal performance gain does not justify the increased memory overhead. Consequently, a batch size of 128 was adopted as the standard configuration for this study.

## 5. Conclusions

This paper proposes a self-supervised cross-domain identification framework that integrates contrastive learning with adversarial domain adaptation. By incorporating a domain discriminator into the contrastive learning architecture, the method aligns the feature distributions of the source and target domains through adversarial training, thereby facilitating the acquisition of domain-invariant features. In domain adaptation tasks across the PLAID and WHITED datasets, this method significantly outperforms traditional supervised approaches when fine-tuned with a small amount of labeled target domain data, which validates its advantages in label-scarce scenarios. Furthermore, in ablation experiments where the encoder is frozen and only the classifier is trained, the features extracted by this method exhibit clearer category separation in t-SNE visualizations, providing further evidence of its superior feature quality and generalization capability.

Despite the significant cross-domain identification performance achieved by the proposed method, three limitations remain. First, constrained by the intersection of categories across cross-domain datasets, the model’s performance was verified on only 11 typical load types, and did not cover all 54 device types in the WHITED dataset; consequently, the model’s applicability in large-scale complex scenarios requires further verification. Second, the model exhibited high sensitivity to label noise, particularly when distinguishing loads with similar features (e.g., Hair Dryers and Heaters), where the impact of incorrect labels on performance was significant. Finally, regarding computational cost, the joint optimization of contrastive learning and adversarial training led to a prolonged offline pre-training duration. Although the online inference latency was low due to model simplification during the inference phase, enabling efficient local online incremental updating and training on resource-constrained edge devices (such as smart meters) remains a critical challenge to be addressed in future work.

Future work will focus on enhancing the method’s identification capability for similar devices in the presence of label noise and exploring lightweight training techniques, thereby further strengthening its applicability and engineering value in real-world household environments.

## Figures and Tables

**Table 1:** Data usage and network structures of each model.

Model	Training Data Source	Training Set Size (Source/Target)	Test Set Size (Target)	Projection Head	Contrastive Learning	Domain Adaptation	Adversarial Training
SimCLR-S	Source domain	22,000/0	3300	Yes	Yes	No	No
SimCLR-M	Source domain +Target domain	11,000/11,000	3300	Yes	Yes	Yes	No
**Proposed**	**Source domain** **+Target domain**	**11,000/11,000**	**3300**	**Yes**	**Yes**	**Yes**	**Yes**

**Table 2:** Model training parameters.

Model	Epoch	Batch Size	Base Encoder		Discriminator	
			Optimizer	lr	Optimizer	lr
SimCLR-S	200	128	Adam	0.0003	\	\
SimCLR-M	200	128	Adam	0.0003	\	\
**Proposed**	**200**	**128**	**Adam**	**0.0003**	**ADAM**	**0.0001**

**Table 3:** Comparison of accuracy across different models.

Model	Appliance											Average
	AC	CFL	Fan	Fridge	Hairdryer	Heater	ILB	Laptop	Microwave	Vacuum	Washing	
SimCLR-S	66.34	75.13	70.35	58.89	75.6	60.74	68.43	77.85	71.27	66.38	66.31	68.8
SimCLR-M	73.83	81.26	78.39	66.61	80.52	68.92	77.25	80.16	75.37	73.72	72.11	75.3
**Proposed**	**82.94**	**90.22**	**86.65**	**79.91**	**87.13**	**80.79**	**87.5**	**86.34**	**87.47**	**86.37**	**83.38**	**85.3**

**Table 4:** Pre-training and fine-tuning hyperparameter settings.

Pretrain	
Optimizer	Adam
Max pretrain epochs	200
Batch size	128
Start learning rate	0.001
Fine-tune	
Batch size	128
Max fine-tune epochs	30
Start learning rate	0.001

**Table 5:** Data usage details.

Stage	Data Source	Samples per Class	Total Samples	Label Usage
Pre-training	Source + Target Domain	2000/class	44,000	Unlabeled
Fine-tuning	Target Domain	20/200/400/600/class	220/2200/4400/6600	Labeled
Validation	Target Domain	500/class	5500	Labeled
Test	Target Domain	1000/class	11,000	Unlabeled

**Table 6:** F1-scores of models under different amounts of labeled training data.

Method	F_Macro_			
	1%	10%	20%	30%
AlexNet	0.2513	0.3508	0.5377	0.7456
VGG16	0.3269	0.4263	0.7782	0.8587
ResNet-18	0.3312	0.5485	0.8242	0.8854
SE-ResNet	0.3395	0.6708	0.8703	0.9127
**Ours**	**0.8449**	**0.9498**	**0.9652**	**0.9787**

**Table 7:** Confusion matrix evaluation metrics of the proposed method at a 30% labeling ratio.

	AC	CFL	Fan	Fridge	Hairdryer	Heater	ILB	Laptop	Microwave	Vacuum	Washing	Average
A	0.990	1	0.998	0.989	0.996	0.998	0.998	0.999	0.998	0.997	0.993	**0.996**
P	0.930	1	1	0.933	0.968	0.992	0.991	1	1	1	0.956	**0.979**
R	0.964	1	0.976	0.948	0.992	0.985	0.987	0.991	0.981	0.967	0.973	**0.979**
F1	0.947	1	0.988	0.941	0.980	0.988	0.989	0.996	0.990	0.983	0.964	**0.979**

Note: Bold values denote the average values of each metric in the table.

**Table 8:** Confusion matrix evaluation metrics of the SE-ResNet method at a 30% labeling ratio.

	AC	CFL	Fan	Fridge	Hairdryer	Heater	ILB	Laptop	Microwave	Vacuum	Washing	Average
A	0.979	0.991	0.981	0.964	0.976	0.978	0.987	0.991	0.994	0.989	0.982	**0.983**
P	0.868	0.940	0.882	0.792	0.855	0.905	0.917	0.981	0.994	0.947	0.990	**0.916**
R	0.925	0.975	0.930	0.846	0.905	0.871	0.948	0.927	0.943	0.938	0.827	**0.912**
F1	0.895	0.957	0.905	0.818	0.879	0.888	0.932	0.953	0.968	0.942	0.901	**0.913**

Note: Bold values denote the average values of each metric in the table.

**Table 9:** F1-scores and standard deviations of the proposed method across five independent experiments.

Label Ratio	Run 1 (%)	Run 2 (%)	Run 3 (%)	Run 4 (%)	Run 5 (%)	Average (%)	Std (%)
1%	84.30	84.65	84.40	84.70	84.40	84.49	**0.175**
10%	94.80	95.15	94.90	95.20	94.85	94.98	**0.182**
20%	96.35	96.70	96.45	96.75	96.35	96.52	**0.192**
30%	97.70	98.05	97.82	98.08	97.70	97.87	**0.185**

Note: Bold values denote the standard deviation (std) in the table.

**Table 10:** Comparison of computational costs of different models.

Model	Parameters (M)	Training Time (h)	Inference Latency (ms/Sample)
ResNet-18	11.2	1.5	4.1
SimCLR-S	11.8	3.5	4.5
**Proposed**	**12.5**	**4.1**	**4.5**

**Table 11:** Comparison of model performance under different key hyperparameters.

Parameter	Setting	Accuracy (%)	F1-Score
Temperature (τ)	0.05	83.2	0.9152
	**0.1 (Ours)**	**85.3**	**0.9498**
	0.5	84.1	0.9234
	1.0	81.5	0.8910
GRL Weight (λ)	0.1	84.5	0.9320
	**1.0 (Ours)**	**85.3**	**0.9498**
Batch Size	64	83.8	0.9210
	**128 (Ours)**	**85.3**	**0.9498**
	256	85.4	0.9501

## Data Availability

The data used in this study are the publicly available PLAID dataset (https://doi.org/10.6084/m9.figshare.10084619) and WHITED dataset (https://www.cs.cit.tum.de/dis/resources/whited/).
